# Supplementary material for: Chemokine expression profile of an innate granuloma
Source: eLife. 2024 Nov 14;13:RP96425. doi: 10.7554/eLife.96425 (PMC11563579; doi:10.7554/eLife.96425)
Supplement: Source code 1. [file elife-96425-code1.zip › eLife-VOR-RA-2024-96425/Streamlined Code.docx]

**Spatial Transcriptomics: Streamlined Code for Analysis Using RStudio**

library(tidyverse)

library(Seurat)

library(RColorBrewer)

library(scales)

df <- readRDS("C:/Users/mergered8_clustree.rds")

Idents(df) <- df$SCT_snn_res.0.9

mylist <- hue_pal()(16)

**Figure 1A,D:**

SpatialDimPlot(df, ncol = 4, pt.size.factor = 1.2, alpha = c(0,0), stroke = 0.25, crop = FALSE, label = FALSE, label.size = 1, label.box = T) & NoLegend()

SpatialDimPlot(df, ncol = 4, pt.size.factor = 1.2, alpha = c(1,1), stroke = 0.25, crop = FALSE, label = FALSE, label.size = 1, label.box = T) & NoLegend()

SpatialDimPlot(df, ncol = 4, pt.size.factor = 1.2, alpha = c(1,1), stroke = 0.25, crop = FALSE, label = FALSE, label.size = 1, label.box = T)

**Figure 1B:**

levels(df) <- c("5","11","9","8","6","0","15","2","1","12","4","14","3","7","13","10")

df[["annotatation"]] <- Idents(df)

Idents(df) <- df$annotatation

DimPlot(df, label = T, cols = c(mylist[6],mylist[12],mylist[10],mylist[9],mylist[7],mylist[1],

mylist[16],mylist[3],mylist[2],mylist[13],mylist[5],mylist[15],

mylist[4],mylist[8],mylist[14],mylist[11])) + NoLegend()

**Figure 1C:**

Idents(df) <- df$SCT_snn_res.0.9

levels(df) <- c("5","11","9","8","6","0","15","2","1","12","4","14","3","7","13","10")

df <- RenameIdents(df, "5" = "NC-C","11" = "NC-P","9" = "CN","8" = "CN-M1", "6"= "CN-M2","0" = "M",

"15" = "OG","2" = "HEP", "1"="HEP", "12"= "HEP","4"= "HEP","14"= "HEP","3"= "HEP","7"= "HEP",

"13" = "EC","10" = "EC")

df[["zones"]] <- Idents(df)

df$library <- factor(df$library, c("1_12_hpi", "7_24_hpi", "9_3_dpi", "10_5_dpi", "11_7_dpi", "12_10dpi", "13_14dpi", "14_21dpi"))

Timepoints <- c("0.5dpi", "1dpi", "3dpi", "5dpi", "7dpi", "10dpi", "14dpi", "21dpi")

levels(df$library) <- Timepoints

CellsbyTime_table <- table(df$library, df$zones)

CellsbyTime_table <- as.data.frame(CellsbyTime_table)

colnames(CellsbyTime_table)

colnames(CellsbyTime_table)[1] <- "Timepoint"

colnames(CellsbyTime_table)[2] <- "Zone"

ByDayPropTable <- CellsbyTime_table %>% group_by(Timepoint) %>% mutate(percent = prop.table(Freq))

Zone_colors <- hue_pal()(9)

dfMinusNonImmune <- subset(df, idents = c("NC-C","NC-P", "CN", "CN-M1", "CN-M2", "M", "OG"))

dfMinusNonImmune7dpi <- subset(dfMinusNonImmune, library != "7dpi")

dfMinusNonImmune7dpi$library <- droplevels(dfMinusNonImmune7dpi$library)

levels(dfMinusNonImmune7dpi$library)

dfMinusNonImmune7dpi$zones <- droplevels(dfMinusNonImmune7dpi$zones)

levels(dfMinusNonImmune7dpi$zones)

ggplot(data = ByDayPropTable, aes(x = Timepoint, y = Zone, size = percent)) +

geom_point(color = "red") +

scale_size(range = c(-.4,10), limits = c(0,1))

CellsbyTime_table <- table(dfMinusNonImmune7dpi$library, dfMinusNonImmune7dpi$zones)

CellsbyTime_table <- as.data.frame(CellsbyTime_table)

colnames(CellsbyTime_table)

colnames(CellsbyTime_table)[1] <- "Timepoint"

colnames(CellsbyTime_table)[2] <- "Zone"

ByDayPropTable <- CellsbyTime_table %>% group_by(Timepoint) %>% mutate(percent = prop.table(Freq))

Zone_colors <- hue_pal()(9)

ggplot(data = ByDayPropTable, aes(x = Timepoint, y = Zone, size = percent)) +

geom_point(color = "red") +

scale_size(range = c(-.4,10), limits = c(0,1))

**Figure 1E; Figure 2; Figure 3; Figure 1 – figure supplement 1C; Figure 4 – figure supplement 1 – figure supplement 6:**

Idents(df) <- df$zones

Idents(df) <- df$Timepoint

SpatialFeaturePlot(df, pt.size.factor = 1.2, alpha = c(1,1), stroke = 0.25, "Ccl2", crop = F, ncol = 4) &

scale_fill_gradientn(colours = rev(brewer.pal(n = 11, name = "RdYlBu")), limits = c(0,3), oob=squish)

**Figure 5A,B:**

Idents(df) <- df$SCT_snn_res.0.9

df[["annotatation"]] <- Idents(df)

df <- RenameIdents(df, "5" = "NC-C","11" = "NC-P","9" = "CN","8"= "CN-M1","6" = "CN-M2", "0" = "M",

"15" = "OG","2" = "HEP0", "1"="HEP1", "12"= "HEP2","4"= "HEP3","14"= "rep HEP","3"= "HEP4","7"= "HEP5",

"13" = "EC1","10" = "EC2")

FeaturePlot(df, c("Cxcl1"), label = T, max.cutoff = 1.5) + scale_colour_gradientn(colours = rev(brewer.pal(n = 7, name = "RdBu"))) + theme(text=element_text(family="Arial"))

**Figure 5C,D:**

df <- RenameIdents(df, "5" = "NC-C","11" = "NC-P","9" = "CN","8"= "CN-M1","6" = "CN-M2", "0" = "M",

"15" = "OG","2" = "HEP0", "1"="HEP1", "12"= "HEP2","4"= "HEP3","14"= "rep HEP","3"= "HEP4","7"= "HEP5",

"13" = "EC1","10" = "EC2")

df[["zones"]] <- Idents(df)

VlnPlot(df, c("Cxcl1"), split.by = "zones", ncol = 1, cols = c(mylist[6],mylist[12],mylist[10],mylist[9],mylist[7],mylist[1], mylist[16],mylist[3],mylist[2],mylist[13],mylist[5],mylist[15],mylist[4],mylist[8],mylist[14],mylist[11]))

**Figure 5E,F:**

df$library <- factor(df$library, c("1_12_hpi", "7_24_hpi", "9_3_dpi", "10_5_dpi", "12_10dpi", "13_14dpi", "14_21dpi"))

Timepoints <- c("0.5dpi", "1dpi", "3dpi", "5dpi", "10dpi", "14dpi", "21dpi")

levels(df$library) <- Timepoints

Idents(df) <- factor(df$library)

df[["Timepoint"]] <- Idents(df)

VlnPlot(df, c("Cxcl1"), split.by = "Timepoint", ncol = 1)

**Figure 1 – figure supplement 1A:**

SpatialFeaturePlot(df, pt.size.factor = 1.2, alpha = c(1,1), stroke = 0.25, features = "nCount_Spatial", crop = F, ncol = 4) &

scale_fill_gradientn(colours = rev(brewer.pal(n = 11, name = "RdYlBu")), limits = c(0,60000), oob=squish)

**Figure 1 – figure supplement 1B:**

VlnPlot(df, features = "nCount_Spatial", pt.size = 0.1, cols = c(mylist[6],mylist[12],mylist[10],mylist[9],mylist[7],mylist[1],

mylist[16],mylist[3],mylist[2],mylist[13],mylist[5],mylist[15],

mylist[4],mylist[8],mylist[14],mylist[11]))

**Table 4:**

PrepSCTFindMarkers(df)

markers <- FindAllMarkers(df, recorrect_umi=FALSE, only.pos = T, min.pct = 0.25, assay = "SCT")

write.csv(markers, "C:/Users/13178/Desktop/myfile.csv")
